# Bagaza Virus in Wild Birds, Portugal, 2021

**DOI:** 10.3201/eid2807.212408

**Published:** 2022-07

**Authors:** João Queirós, Sílvia C. Barros, Alberto Sánchez-Cano, Ana Margarida Henriques, Teresa Fagulha, Fábio Abade dos Santos, Margarida D. Duarte, Catarina Fontoura-Gonçalves, David Gonçalves, Marisa Rodrigues, Teresa Cardona Cabrera, Isabel G. Fernández de Mera, Christian Gortazar, Ursula Höfle, Paulo Célio Alves

**Affiliations:** Universidade do Porto Centro de Investigação em Biodiversidade e Recursos Genéticos, Vairão, Portugal (J. Queirós, C. Fontoura-Gonçalves, D. Gonçalves, M. Rodrigues, P.C. Alves);; Estação Biológica de Mértola, Mértola, Portugal (J. Queirós, D. Gonçalves, M. Rodrigues, P.C. Alves);; Universidade do Porto, Porto, Portugal (J. Queirós, D. Gonçalves, P.C. Alves);; Instituto Nacional de Investigação Agrária e Veterinária, Oeiras, Portugal (S.C. Barros, A.M. Henriques, T. Fagulha, F.A. Abade dos Santos, M.D. Duarte);; Instituto de Investigación en Recursos Cinegéticos, Ciudad Real, Spain (A. Sánchez-Cano, T.C. Cabrera, I.G. Fernández de Mera, C. Gortazar, U. Höfle)

**Keywords:** Bagaza virus, birds, Portugal, flavivirus, BAGV, outbreak, *Alectoris rufa*, *Emberiza calandra*, Portugal, viruses

## Abstract

Bagaza virus emerged in Spain in 2010 and was not reported in other countries in Europe until 2021, when the virus was detected by molecular methods in a corn bunting and several red-legged partridges in Portugal. Sequencing revealed high similarity between the 2021 strains from Portugal and the 2010 strains from Spain.

Bagaza virus (BAGV) is a single-stranded, positive-sense RNA virus. The virus belongs to the mosquitoborne cluster of the genus *Flavivirus,* family *Flaviviridae,* which includes such other emerging pathogens as West Nile, Japanese encephalitis, dengue, Zika, and yellow fever viruses, all of which are associated with neurologic disease in animals and humans and have zoonotic potential (*1*). BAGV was first isolated in 1966 from a pool of *Culex* species mosquitoes in the Bagaza District of Central African Republic and was detected subsequently in several species of mosquitoes. The first BAGV-associated deaths in vertebrates were detected in Spain, in 2010, in red-legged partridges (*Alectoris rufa*) and ring-necked pheasants (*Phasianus colchicus*) (*2*) and then, in 2016, in Himalayan monal pheasants (*Lophophorus impejanus*) in South Africa (*3*). 

BAGV infection causes neurologic disease in red-legged partridges, gray partridges (*Perdix perdix*), ring-necked pheasants, and, to a lesser degree, in common wood pigeons (*Columba palumbus*) (*1*–*6*). Estimated mortality rates range from 23% to 30% in naturally and experimentally infected red-legged partridges (*5*,*7*); rates are higher (up to 40%) in experimentally infected gray partridges (*6*) and lower rates in pheasants and columbiformes (*4*,*7*). We describe a BAGV outbreak in Portugal in autumn 2021, associated with abnormal fatalities in red-legged partridges and 1 corn bunting (*Emberiza calandra*).

On September 1, 2021, three red-legged partridges were found dead in Serpa, southern Portugal. From September through mid-October, 9 partridges and 1 corn bunting were found dead in the same area (Appendix Table). Local reports emerged of partridges displaying neurologic signs compatible with potential viral infection, such as disorientation and motor incoordination. Twelve of the 13 birds were necropsied. Laboratory examinations and preliminary diagnoses were conducted at the Research Institute in Hunting Resources (Ciudad Real, Spain) and at the Center for Research on Biodiversity and Genetic Resources (*InBIO* Laboratório Associado, Vairão, Portugal). Official diagnosis was determined at the National Institute of Agrarian and Veterinary Research, I.P. (Lisbon, Portugal). Growing feathers were collected from 30 partridges live-trapped in the same area on October 3.

Researchers conducted molecular detection by using RNA extracted from various sampling points (feather pulp, brain, heart, kidney, spleen, and intestine) and followed 2 strategies targeting different regions of the BAGV genome (nonstructural 2b, nonstructural 5 [NS5], and 3′ nontranslated region) (Appendix Table); first, a duplex quantitative reverse transcription PCR (RT-PCR) for the simultaneous and differential detection of Japanese encephalitis and Ntaya flavivirus serocomplexes (*8*), and second, a uniplex quantitative RT-PCR specific for the NS5 coding region of BAGV (*9*). The researchers used conventional nested RT-PCR for sequencing to target part of the NS5 gene (*10*) and an in-house RT-PCR (developed at the National Institute of Agrarian and Veterinary Research) to target part of the NS2b gene (Appendix Table).

Out of the 12 necropsied birds, 8 red-legged partridges and 1 corn bunting (75%) tested positive for BAGV, as did 4 of 30 live-captured red-legged partridges (13.3%) (Appendix Table). The 108 bp sequences obtained from duplex quantitative RT-PCR from partridge 9 and the corn bunting showed 100% similarity with the 3′ nontranslated region of the BAGV reference strain (GenBank accession no. HQ644143) detected in the 2010 outbreak in Spain (Appendix Table). In comparing the NS5 regions, researchers found very high similarities with HQ644143 in the 110 base pair sequences obtained from 6 partridges by nested RT-PCR (99.1%) and in the 171 base pair sequences taken from 2 partridges by RT-PCR (98.8%).

Upon necropsy, all birds were in good body condition, suggesting an acute disease course. Histopathology, albeit hampered by autolysis and freezing artifacts, revealed lymphoid depletion in the spleen and severe congestion, moderate to abundant diffuse mononuclear inflammatory infiltrates, and focal necrosis in all tissues. The heart, brain, kidney, and liver were the most affected organs (Figure).

**Figure:**
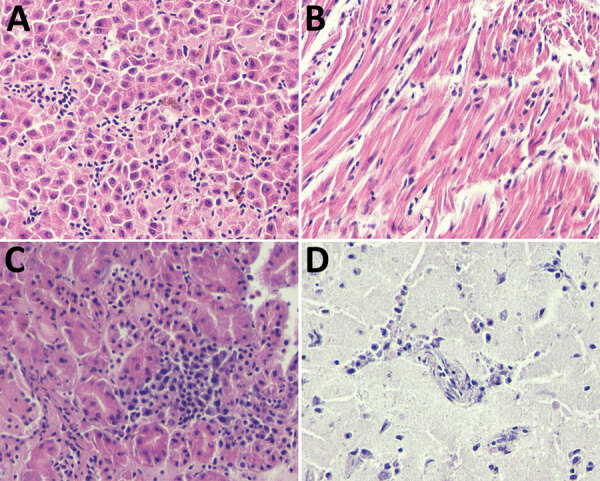
Microscopic lesions caused by Bagaza virus infection in liver, heart, kidney, and brain tissue of red-legged partridges (*Alectoris rufa*), Portugal, 2021. A) In liver, congestion, hemozoin presence in Kupffer cells, focal hepatocyte necrosis, and a moderate mononuclear infiltrate are visible despite some freezing artifacts. B) In heart, congestion, hemorrhage, edema, degeneration of myofibers of the myocardium, and endothelial swelling and moderate to abundant diffuse mononuclear infiltrates are visible. C) In kidney, tubulointerstitial nephritis characterized by congestion, hemorrhage necrosis of proximal convoluted tubular epithelium, and diffuse moderate to abundant mononuclear inflammatory infiltrate are visible. D) In brain, mild nonpurulent encephalitis with congestion, mononuclear cell extravasation, and endothelial cell swelling are visible. Hematoxilin eosin staining; original magnification ×400.

This work confirms BAGV emergence in Portugal, in autumn 2021, associated with abnormal fatalities in red-legged partridges. Active circulation of BAGV was also evidenced in the studied region, where 13.3% of live-captured red-legged partridges testing positive for BAGV, even though ecologic and demographic studies are required to determine the extent and magnitude of the outbreak. Substantial population decline in the red-legged partridge can be anticipated in this region of Portugal on the basis of the mortality rate previously estimated for this species (*4*,*7*). The fatal case in a songbird, the corn bunting, suggests that BAGV might have a broader spectrum and effect in wild bird species. This finding, combined with the small size of the analyzed sequences, suggests the need for further research to identify the vectors for BAGV in Portugal and their role in the epidemiology of the disease, and elucidate the phylogenetic relationships between the 2021 strains in Portugal and 2010 strains in Spain against known BAGV strains. 

No conclusions can be made from this research regarding the origin of this infection. However, the introduction of the virus in Portugal might be linked to persistence of the disease and migration of infected wild birds from North Africa or Spain.

Appendix Additional information about bagaza virus in wild birds, Portugal, 2021.
