# Supplementary material for: Bagaza Virus in Wild Birds, Portugal, 2021
Source: Emerg Infect Dis. 2022 Jul;28(7):1504–6. doi: 10.3201/eid2807.212408 (PMC9239872; doi:10.3201/eid2807.212408)
Supplement: Appendix — Additional information about bagaza virus in wild birds, Portugal, 2021. [file 21-2408-Techapp-s1.pdf]

Article DOI: http://doi.org/10.3201/eid2807.212408

Bagaza Virus in Wild Birds, Portugal, 2021

Appendix

Appendix Table. Bagaza virus–positive specimens analyzed in a study of wild birds in Portugal, 2021*

						Real time RT-PCR (RT-qPCR)								Pan-Flavivirus conventional RT-PCR	
						Duplex RT-qPCR[1] (3′NTR, 172 bp amplicon)				Uniplex RT-qPCR[2] (NS5, 61pb amplicon)				nested RT-PCR[3] (NS5, 143 bp amplicon)	RT-PCR[4] (NS2b, 209 bp amplicon)
Code	Species	Status	Collection date	Sex	Age class	Feather	Brain	Kidney	Spleen	Heart	Kidney	Brain	Intestine	Feather/spleen	Heart
Partridge 1	*A. rufa*	Found dead	01/09/21	Male	Adult	+	–	–	–	nt	nt	nt	nt	+(#)	nt
Partridge 2	*A. rufa*	Found dead	01/09/21	Male	Adult	–	–	–	+	nt	nt	nt	nt	+(#)	nt
Partridge 3	*A. rufa*	Found dead	28/09/21	Female	Juvenile	+	+	–	nt	nt	nt	nt	nt	–	nt
Partridge 4	*A. rufa*	Found dead	29/09/21	Female	Juvenile	+	+	–	nt	nt	nt	nt	nt	+(#)	nt
Corn Bunting	*E. calandra*	Found dead	09/10/21	Unknown	Unknown	–	–	–	+(#)	nt	nt	nt	nt	–	nt
Partridge 7	*A. rufa*	Found dead	13/10/21	Male	Adult	nt	nt	nt	nt	+	+	+	+	nt	+(#)
Partridge 8	*A. rufa*	Found dead	13/10/21	Male	Adult	nt	nt	nt	nt	+	+	+	+	nt	+(#)
Partridge 9	*A. rufa*	Found dead	13/10/21	Female	Juvenile	+(#)	+(#)	+	nt	nt	nt	nt	nt	+(#)	nt
Partridge 11	*A. rufa*	Found dead	14/10/21	Female	Juvenile	+	+	+	nt	nt	nt	nt	nt	+(#)	nt
Partridge 14	*A. rufa*	Live-trapped	03/10/21	Male	Adult	+	na	na	na	na	na	na	na	+(#)	na
Partridge 16	*A. rufa*	Live-trapped	03/10/21	Male	Adult	+	na	na	na	na	na	na	na	–	na
Partridge 33	*A. rufa*	Live-trapped	03/10/21	Male	Adult	+	na	na	na	na	na	na	na	–	na
Partridge 37	*A. rufa*	Live-trapped	03/10/21	Male	Juvenile	+	na	na	na	na	na	na	na	–	na

*+, positive; -, negative; nt, not tested; na, not applicable; # - samples for which successful sequences were obtained. 1- dRT-qPCR developed for the detection of Japanese Encephalitis virus and virus from the Ntaya serocomplex (108 nt-long sequences obtained from Partrdige 9 and corn bunting showed 100% similarity with BAGV sequence HQ644143, position 10,510 to 10,680); 2- RT-qPCR developed for the specific detection of BAGV (not sequenced), 3- nested RT-PCR developed by Sánchez-Seco et al. (2005) for the detection of *Flavivirus* (110 nt-long sequences obtained from Partridge 1, 2, 4, 11 and 14 showed 99.1% similarity with BAGV sequence HQ644143, position 8,996 to 9,139); 4- RT-PCR, developed by the INIAV team (unpublished, 171 nt-long sequences obtained from Partridge 7 and 8 showed 98.8% similarity with BAGV sequence HQ644143). Sizes of the sequences do not include the primers' annealing sequences. HQ644143 is the reference BAGV genome strain from the 2010 outbreak in Spain.
